# Histopathological lesions and exposure to *Plasmodium falciparum* infections in the placenta increases the risk of preeclampsia among pregnant women

**DOI:** 10.1038/s41598-020-64736-4

**Published:** 2020-05-19

**Authors:** Dorotheah Obiri, Isaac Joe Erskine, Daniel Oduro, Kwadwo Asamoah Kusi, Jones Amponsah, Ben Adu Gyan, Kwame Adu-Bonsaffoh, Michael Fokuo Ofori

**Affiliations:** 1 West African Centre for Cell Biology of Infectious Pathogens (WACCBIP), Department of Biochemistry, Cell and Molecular Biology, College of Basic and Applied Sciences, University of Ghana, Legon, P.O. Box LG54, Accra, Ghana; 2 Immunology Department, Noguchi Memorial Institute for Medical Research, College of Health Sciences, University of Ghana, Legon, P.O. Box LG581, Accra, Ghana; 3 Department of Pathology, Korle-Bu Teaching Hospital, Accra, Ghana; 4 Department of Animal Biology and Conservation Science, College of Basic and Applied Sciences, University of Ghana, Legon, P.O. Box LG67, Accra, Ghana; 5 Weill Cornell Medicine, Cornell University, New York, United States of America; 6 Department of Obstetrics and Gynaecology, University of Ghana Medical School, Accra, Ghana

**Keywords:** Diseases, Health care, Pathogenesis, Risk factors

## Abstract

Preeclampsia (PE) is a placental disorder with different phenotypic presentations. In malaria-endemic regions, high incidence of PE is reported, with debilitating foeto-maternal effects, particularly among primigravid women. However, the relationship between placental pathology and *Plasmodium falciparum* infection in the placenta with PE is underexplored. Placentas from 134 pregnant women were examined after delivery for pathological lesions and placental malaria (PM). They comprised of 69 women without PE (non-PE group) and 65 women diagnosed with PE (PE group). The presence of placental pathology increased the risk of PE, with particular reference to syncytial knots. Placental malaria was 64 (48.1%) and 21 (15.8%) respectively for active and past infections and these proportions were significantly higher in the PE group compared to the non-PE group. Further multivariate analyses showed placental pathology (adjusted (aOR) 3.0, 95% CI = 1.2–7.5), active PM (aOR 6.7, 95% CI = 2.3–19.1), past PM (aOR 12.4, 95% CI = 3.0–51.0) and primigravidity (aOR 6.6, 95% CI 2.4–18.2) to be associated with PE. Our findings suggest that placental histological changes and PM are independent risk factors for PE particularly in primigravida. These findings might improve the management of PE in malaria-endemic regions.

## Introduction

The maternal syndrome of preeclampsia (PE) complicates 2–10% of pregnancies worldwide^1^ and contributes significantly to maternal and foetal morbidity and mortality^2^. It is clinically characterised by the new onset of hypertension and proteinuria after 20 weeks of gestation in previously normotensive women^3^. However, the risk is also increased in women with chronic hypertension and other metabolic conditions such as diabetes^4^. In Ghana, maternal mortality due to PE and other hypertensive disorders in pregnancy has doubled over the past decade (9% in 2007 to 18% in 2017)^5^. Subsequently, these disorders have emerged as the leading cause of maternal mortality in the tertiary centres of the country^6,7^.

While the search for aetiological links to PE continues, significant advancements have been made in understanding the pathophysiologic role of the placenta in the development of PE.

Histopathological examination of placental biopsies from pregnancies complicated by PE and foetal growth restriction revealed inadequate trophoblast invasion and impaired remodelling of maternal spiral arteries^8,9^. Further on, the two-stage model of PE by Robert and Hubel^10^ suggests a causal association between placental insufficiencies (stage 1) and the development of clinical symptoms (stage 2). Poor placental perfusion has been associated with placental histopathological lesions such as infarcts, increase in syncytial knots, fibrin deposits, atherosis of the arterial wall, accelerated villous maturation and calcifications^11–16^. These changes result from ischaemic and hypoxic mechanisms secondary to poor perfusion^17^. Additionally, variable effects on the clinical severity of PE and foetal outcomes have been shown to implicate several placental factors in the pathogenesis of PE^14,16^.

In malaria-endemic regions, women exposed to placental parasites could be at an increased risk of PE compared to those in non-malarious regions. Although there are conflicting reports, most studies have confirmed this theory^18–21^. Sequestration of infected erythrocytes in the placenta is the hallmark of placental malaria pathogenesis and its effects have been associated with the pathogenesis of PE^22,23^. Consequential adverse effects such as low birth weight and foetal death resulting from placental pathological alterations have been reported in both conditions^24–27^. These alterations, such as excessive fibrinoid deposits associated with syncytiotrophoblast damage, and ultra-structural damage lead to basal lamina thickening in placentas affected by placental malaria (PM)^28^. Immunopathologic processes are also related to these placental changes. Specifically, heightened inflammation has been reported in murine model of PM leading to tissue disorganisation, reduced vascular spaces and blood supply in placental tissues^29^. Markers of hypoxia such as hypoxia-inducible factors and tissue damage are also increased in women with sub-microscopic PM infections^30^.

Over the years, the effects of placental changes during PM have focused mainly on foetal outcome, maternal anaemia and death. Maternal death due to PM is believed to be indirect. Besides, most women with placental parasite infections are either asymptomatic and or lack sensitivity to peripheral blood test^31^. The silent placental infection may be associated with pathological changes, including localised placental inflammation, which may compromise placental function. In effect, PM may induce or exacerbate pregnancy-associated syndrome such as PE. The possible link between placental pathological lesions, PM and the outcome of PE has been investigated in this study by testing the hypothesis that more women with PM and its associated histological lesions suffer from the PE syndrome.

## Results

### Characteristics and perinatal outcome of study participants

A total of 134 placentas were sampled at delivery comprising 65 from women diagnosed with PE (PE group) and 69 without PE (non-PE group) (Table 1). The age of the pregnant women ranged between 18–42 years and was similar between the two groups. Body mass index (BMI) and intermittent preventive treatment in pregnancy (IPTp) coverage were also similar between the PE and the non-PE groups (*P* > 0.05 in all cases). Most women (91%) received between one to four doses of IPTp during gestation. Gravidity was different between the groups (*P* = 0. 003). Systolic blood pressure (SBP) and diastolic blood pressure (DBP) at sampling were significantly higher in the PE (mean SBP = 150.7 ± 18.1, mean DBP = 95.7 ± 14.3; *P* < 0.0001) compared to non-PE group (mean SBP 122.2 ± 14.2, mean DBP = 75.5 ± 8.8; *P* < 0.0001). For perinatal outcome, the PE group had significantly lower gestational age at delivery (36.2 ± 4 weeks), vaginal deliveries (23.1% of 65), and birth weight (2.4 ± 1 kg) compared to the non-PE group [(gestational age at delivery 39.5 ± 2 weeks, *P* < 0.0001); vaginal deliveries (100% of 69, *P* < 0.0001); birth weight (3.2 ± 0.5 kg, *P* < 0.0001) (Table 1). Apgar score which measures the physical characteristics of the newborn was low resulting in a higher number of neonatal intensive care unit (NICU) admissions among the PE group deliveries (57.8% of 65, *P* < 0.0001).

**Table 1:** Demographic and clinical characteristics among women diagnosed with or without preeclampsia.

Characteristic	Non-PE n = 69	PE n = 65	Total n = 134	P-value
Maternal age (years)^#^	29 ± 6	28 ± 6	28 ± 6	0.27
Primigravid^*^	16 (23.2%)	32 (49.2%)	48 (35.8%)	0.003
Multigravid^*^	53 (76.8%)	33 (50.8%)	86 (64.2%)	
Mean BMI (kg/m^2^)^#^	26 ± 4.9	27.7 ± 5.6	26.8 ± 5.3	0.08
Sampling Systolic BP (mmHg)^#^	122.2 ± 14.2	150.7 ± 18.1	136.0 ± 21.6	<0.0001
Sampling Diastolic BP (mmHg)^#^	75.5 ± 8.8	95.7 ± 14.3	85.2 ± 15.5	<0.0001
IPTp use^*^				
Dose 4	1 (1.5%)	2 (3.4%)	3 (2.4%)	0.78
Dose 3	36 (55.4%)	27 (46.6%)	63 (51.2%)	
Dose 2	10 (15.4%)	13 (22.4%)	23 (18.7%)	
Dose 1	12 (18.5%)	11 (19.0%)	25 (18.7%)	
Dose 0	6 (9.2%)	5 (8.6%)	11 (8.9%)	
No record	4	7	11	
Delivery age (weeks)^#^	39.5 ± 2	36.2 ± 4	37.9 ± 4	<0.0001
Mode of delivery^*^				
Vaginal	69 (100%)	15 (23.1%)	84 (62.7%)	<0.0001
C section	0	50 (76.9%)	50 (37.3%)	
Preterm delivery^*^	3 (4.3%)	30 (46.3%)	33 (24.6%)	<0.0001
Birth weight (kg)^#^	3.2 ± 0.5	2.4 ± 1.0	2.8 ± 0.8	<0.0001
Apgar at 1 min^#^	7.1 (±1.1)	6.3 (±1.3)	6.7 (±1.3)	<0.002
Apgar at 5 min^#^	8.3 (±0.9)	7.6 (±1.2)	8.0 (±1.1)	<0.001
**Sex of baby**^a*^				
Male	33 (47.8%)	30 (46.9%)	63 (47.4%)	1.00
Female	36 (52.2%)	34 (53.1%)	70 (52.6%)	
**NICU admission**^b*^				
Yes	7 (10.1%)	37 (57.8%)	44 (33.1%)	<0.0001
No	62 (89.9%)	27 (42.2%)	89 (66.9%)	

^#^Mean ± SD; ^*^n = %; ^a^ n = 64 (PE group); ^b^n = 68 (both non-PE and PE groups) and PE NICU admission; BMI = Body mass index; BP = Blood pressure; IPTp = Intermittent preventive treatment in pregnancy; NICU = Neonatal intensive care unit. *P*-values were generated using Student’s t-test for continuous data and Fisher’s exact test for categorical data.

### Placental histological findings in women diagnosed with and without preeclampsia

**Figure 1:**
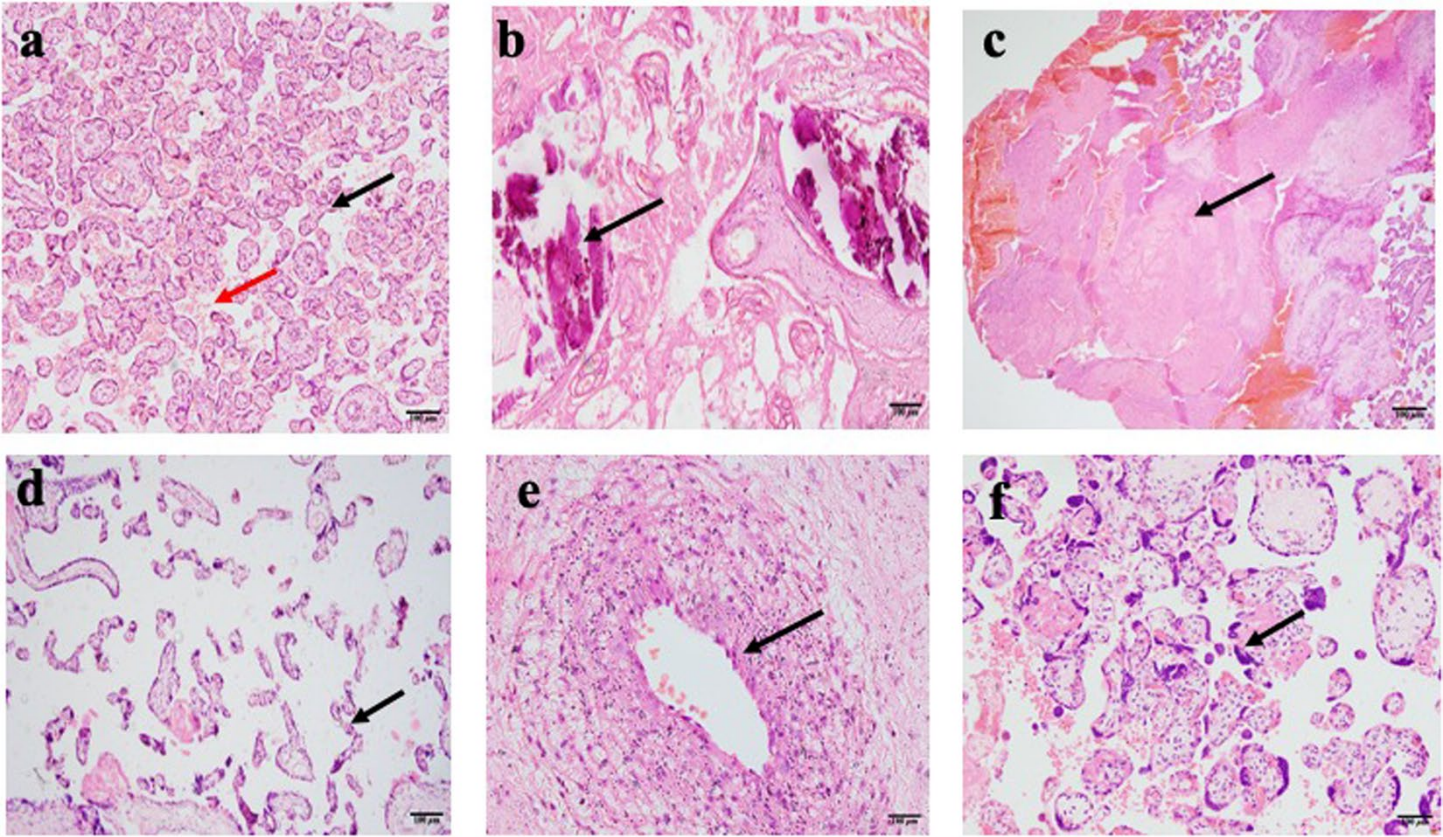
Photomicrographs of placental changes observed among pregnant women from the study. (**a**) A non-PE placenta showing normal villi (black arrow) and intervillous spaces (red arrow). (**b**) Calcifications (black arrow) in a 33-week old PE placenta. (**c**) Infarction (coagulative necrosis) of large area of the placenta from ischaemia (black arrow) in 29-week placenta with intrauterine foetal death). (**d**) Accelerated villous maturation (thin finger-like or slender villi with reduced branching). (**e**) Atherosis showing accumulation of lipid laden macrophages within sub-endothelial area of arterial wall. (**f**) Increased syncytial knots showing densely stained and closely packed nuclei.

**Figure 2:**
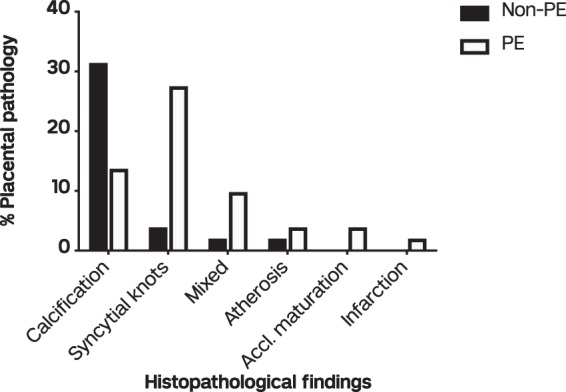
Percentage of histological findings in normal and pathologic placentas. Bars represent non-PE (white bars) and PE (dark bars) women. Mixed = 2 or more placental abnormalities; accl. maturation = accelerated villous maturation.

Unique pathological features were observed and reported by histology from the placentas of non-PE and PE pregnancies. Of the 134 placentas, 51 (38.1%) showed histopathologic lesions and this comprised 20 (29% of 69) in the non-PE group and 31 (47.7% of 65) in the PE group. Calcifications, syncytial knots, infarctions, atherosis, accelerated maturation of villi and a combination of 2 or more of these pathologies (mixed) were the major changes observed in the sampled placentas (Fig. 1). The percentage of placentas with calcifications was pronounced in the non-PE group (31.4% of 51) compared to the PE group (13.7% of 51). Syncytial knots were prominent in the PE group (27.5% of 51) than the non-PE group (3.9% of 51). The mixed group comprising 2 or more observed pathologies within a placenta together with atherosis were not different between the non-PE and PE groups. Accelerated villous maturation and infarctions were observed in the PE group only (Fig. 2). The placental pathologic effects on gravidity and perinatal outcomes were assessed in PE and non-PE pregnancies. Percentage pathology by gravidity is shown in Supplementary Fig. S1a. In the PE group, women with both normal and pathologic placentas had a lower mean delivery age compared to the non-PE group (Supplementary Fig. S1b). Similarly, the mean birth weight of babies delivered to PE women with both normal and pathologic placentas was lower compared to the mean birth weight from the non-PE group (Supplementary Fig. S1c).

### Risk factors associated with pathology in non-preeclamptic and preeclamptic pregnancies

Generally, placental pathology was significantly associated with PE. Presence of placental pathology increased the odds of PE (odds ratio (OR) 2.2, 95% CI 1.1–4.6; *P* = 0.027). Syncytial knots were the only specific pathology that increased the risk of PE (OR 10.1, 95% CI 2.2–47.3; *P* = 0.003). Calcification, mixed pathology, infarction, accelerated villous maturation and atherosis did not show significant effects. Primigravidity increased the odds of developing PE in both the univariate (OR: 3.0, 95% CI (1.5–6.3), *P* = 0.003 respectively) and multivariate (OR: 4.5, 95% CI (2.0–10.7), *P* < 0.0001) analyses (Supplementary Table S1).

### *Plasmodium falciparum* placental exposure and the risk of preeclampsia

**Figure 3:**
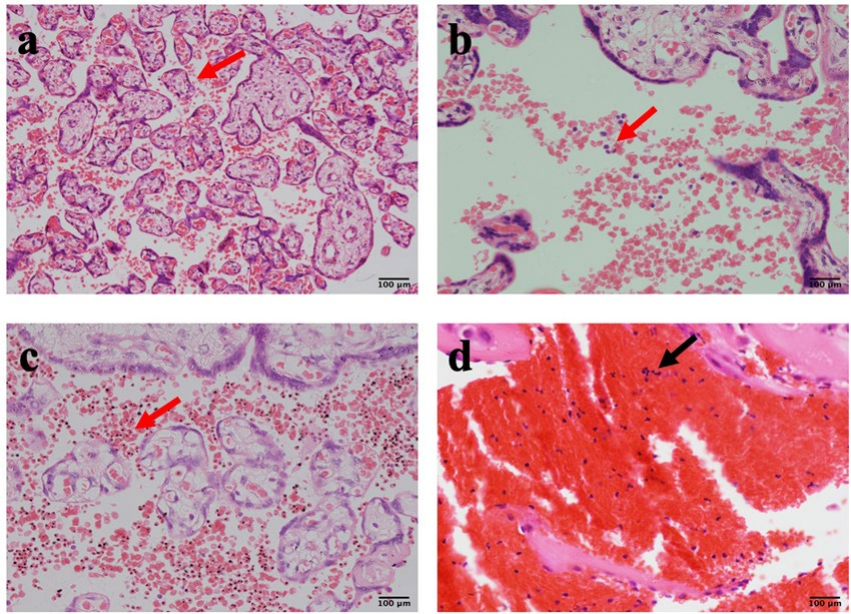
Placental infections observed among pregnant women from the study. (**a**) Normal placental architecture with uninfected RBCs in the intervillous spaces. (**b**) Acute malaria infection with only parasitized red cells and no parasite pigments (**c**) Chronic malaria infection with parasites and parasite pigments present in red cells in a 38-week placenta (**d**) Past malaria infection showing pigments in fibrin.

**Figure 4:**
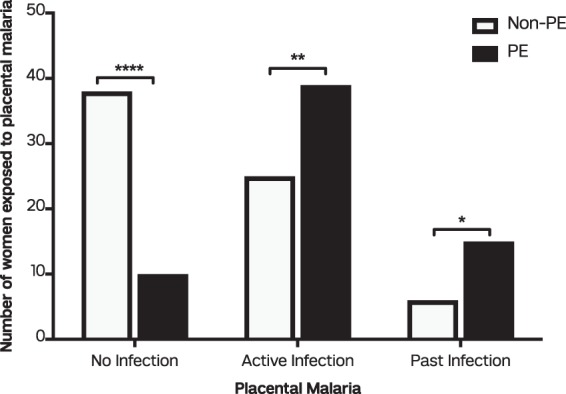
Measure of exposure to placental malaria in women diagnosed with non-PE (white bars) and PE (dark bars). Data presented as proportions between active, past and no infection in non-PE and PE placentas by Fisher’s exact test. ^*^(*P* < 0.05), ^**^(*P* < 0.01); ^****^(*P* < 0.0001).

A total of 133 placentas were evaluable for PM. The classification guide by Bulmer *et al*.^32^, was used to score the absence of infection, acute, chronic and past infections for all the 133 placentas (Supplementary Table S2 and Fig. 3). Overall, 64 (48.1% of 133) placentas had active parasites (acute and chronic infections), 21(15.8% of 133) with past infections and 48 (36.1% of 133) with no infections (Fig. 4). A higher proportion of active and past placental malaria were observed in women diagnosed PE (61.0% and 23.4% of 64 respectively) compared to non-PE diagnosed women (36.2% and 8.7% of 69 respectively). Women with no parasite infections were 10 (15.6% of 64) in PE diagnosed placentas and 38 (55.1% of 69) in non-PE diagnosed placentas.

### Placental exposure, maternal and pregnancy outcomes in preeclampsia

Placental malaria was further evaluated based on gravidity (Table 2). At delivery, the presence of active and past PM was assessed against placentas without parasite infections. In the primigravid women (n = 47), 19 active placental infections (82.6% of 23), 5 past placental infections (71.4% of 7) and 7 with no placental infections (41.2% of 17) was reported for the PE group. In the non-PE group, 4 active placental infections (17.4% of 24), 2 past placental infections (28.6% of 7) and 10 with no placental infections (58.8% of 17; *P* = 0.02). In the multigravid women (n = 86), 20 active placental infections (48.8% of 41), 10 past placental infections (71.4% of 14) and 3 with no placental infections (9.7% of 31) was observed in PE placentas. Placentas from non-PE woman had 21active placental infections (51.2% of 41), 4 past placental infections (28.6% of 14) and 28 with no placental infections (90.3% of 31). The maternal age at delivery was significantly lower in the PE women with active infections (35.7 ± 3.98 weeks) compared to non-PE women (39.4 ± 1.60 weeks; *P* = 0.0002). However, the mean delivery age in women with past infections or no infections did not differ between the groups. (Supplementary Fig. S2a). Also, the mean birth weight of babies was significantly lower amongst PE women with active (2.4 ± 0.96 kg) and past infection (2.4 ± 0.86 kg) compared to those in non-PE women (active infections 3.2 ± 0.50 kg, *P* = 0.0003; past infections 3.3 ± 0.29 kg, *P* = 0.03) (Supplementary Fig. S2b).

**Table 2:** Distribution of parasite infection status by gravidity between study groups.

	No Infection n (%)	Active Infection n (%)	Past-Infection n (%)	Total n (%)	*P*-value
**Primigravid**					
Non-PE	10 (58.8)	4 (17.4)	2 (28.6)	16 (32.0)	0.02
PE	7 (41.2)	19 (82.6)	5 (71.4)	31 (68.0)	
**Multigravid**					
Non-PE	28 (90.3)	21 (51.2)	4 (28.6)	53 (58.9)	<0.0001
PE	3 (9.7)	20 (48.8)	10 (71.4)	33 (41.1)	
Total	48	64	21	133	

Data presented as proportions between non-PE and PE groups in primigravid and multigravid women. P-value obtained by Fisher’s exact test.

### Placental malaria, pathology and pregnancy factors as predictors of preeclampsia

The study compared placental infections, pathology and other significant maternal risk factors for PE development using a logistic regression model (Table 3). The risk of PE was increased in women with active and past placental infections [odds ratio: 5.9 (2.6–14.0), *P* < 0.0001 and 9.5 (2.9–30.8), *P* < 0.0001 respectively]. In a multivariate analysis, the risk of PE was still increased in women with active or past placental infections [adjusted odds ratio: 6.7 (2.3–19.1), *P* < 0.0001; 12.4 (3.0–51.0), *P* < 0.0001 respectively]. This risk was further increased in primigravid women [adjusted odds ratio: 6.6 (2.4–18.2), *P* < 0.0.0001] with placental pathological lesions [adjusted odds ratio: 3.0 (1.2–7.5), *P* = 0.019].

**Table 3:** Crude odds ratio (OR) and adjusted odds ratio (AOR) for factors associated with placental malaria and the risk of PE.

	OR (95% CI)	*P*-value	AOR (95% CI)	*P*-value
**PM**				
No Infection	Ref	Ref	Ref	
Active Infection	5.9 (2.6–14.0)	<0.0001	6.7 (2.3–19.1)	<0.0001
Past Infection	9.5 (2.9–30.8)	<0.0001	12.4 (3.0–51.0)	<0.0001
**Pathology**				
No	Ref	Ref	Ref	
Yes	2.2 (1.1–4.6)	0.027	3.0 (1.2–7.5)	0.019
1^st^ visit SBP (mmHg)	1.0 (1.0–1.1)	0.010	1.04 (1.0–1.1)	0.083
1^st^ visit DBP (mmHg)	1.0 (1.0–1.1)	0.042	1.0 (0.9–1.1)	0.65
**Gravidity**				
Multigravid	Ref	Ref	Ref	
Primigravid	3.0 (1.5–6.3)	0.003	6.6 (2.4–18.2)	<0.0001

Logistic regression model used. *P* value is significant if <0.05. PM = placental malaria, PE = preeclampsia, SBP and DBP = systolic and diastolic blood pressure respectively.

## Discussion

Placental examination is an important, non-invasive means to continuously decipher pathological conditions that improve maternal and foetal health. Therefore, histological examination of placentas after delivery from PE and non-PE diagnosed women in a malaria-endemic region will provide further insight into the pathophysiology of the disorder. From the study, histological alterations occurred more frequently in PE placentas than non-PE placentas. This reflects the placental origin and the pathophysiologic involvement of the placenta in PE pathogenesis. Reports of placental alterations in PE may either be specific^33^ or non-specific^17^ to PE suggesting that these alterations may contribute either directly or indirectly to PE pathogenesis.

Of the specific alterations associated with PE in this study, syncytial knots were identified as the main contributor to placental pathology in women diagnosed with PE. It was about ten-fold more frequent in PE than non-PE placentas. These aggregates of syncytial nuclei are associated with conditions of uteroplacental malperfusion^34^ and hypoxia^35^ such as PE. The syncytiotrophoblasts mainly serve as a protective barrier against harmful effects to the foetus as well as a foeto-maternal exchange portal for nutrients and waste products. Functional deficits of the syncytiotrophoblasts result in the formation of these knots which support the ‘Tenney-Parker’ changes of the placenta used as an index of well-being^36–39^. Our data also confirm findings from a previous report of excessive syncytial knots in PE placentas compared to normotensive women^39^. Increased syncytial knots have also been associated with PE severity^39,40^. The biological inference here is that increased syncytial knots may result in sporadic release of factors into the localized placental environment that cause further damage to the placenta. Bioactive substances released such as soluble vascular endothelial growth factor receptor one (sVEGFR1) may be exported into maternal circulation to cause clinical effects of PE.

Accelerated villous maturations and infarctions were reported in the PE group only hence statistical significance could not be established. These findings which have been shown elsewhere to increase the risk of PE^16^ clearly emphasizes PE as a syndrome of placental dysfunction. Calcifications, atherosis and presence of mixed pathologies were not different between the groups. Although these are biologically significant pathologic alterations in PE pregnancies, literature reports a discordance in their pathophysiologic ramifications^11,15^. Despite these reports, the overall association between placental pathology and PE as shown in this study suggests that these placental lesions may have a contributory effect to the PE syndrome.

Maternal risk factors for PE such as twin gestation, chronic hypertension and diabetes were excluded at recruitment to limit confounders. Other risk factors such as high BMI were not different between the PE and non-PE groups as shown in the demographic characteristics. Primigravidity and blood pressure were independent risk factors for PE in the univariate analysis. These were also evident in a multivariate analysis that included the presence of placental pathology except diastolic blood pressure at booking. Increased blood pressure has been linked to the development of hypertensive disorders in pregnancy (HDP)^41^. Krielessi correlated the extent of placental lesions with the level of hypertensive disorders and found that extensive placental lesions were associated with a higher level of hypertension^42^. However high blood pressure alone, is indicative of gestational hypertension, a different phenotype from PE and may not present with placental pathology as reported elsewhere^43,44^. Primigravidity is also a known risk factor for PE^45,46^. Our study has shown placental pathology, primigravidity but not diastolic blood pressure at booking as contributors to PE and these may discriminate it from other HDP. Our data also support earlier findings regarding adverse pregnancy and foetal outcomes such as low birth weight and pre-term birth associated with PE.

Malaria endemicity may be an added risk factor for PE pathogenesis as demonstrated in earlier studies^18,20,21,47^. The histological grading system for PM showed a high exposure rate in PE than non-PE women. Consistent with earlier studies, PM is an independent risk factor for PE with adverse outcomes such as low birth weight. Approximately nine and six fold increases in the risk for PE were observed for past and active parasite infections respectively, with absence of infections as a reference. In a similar study in Sudan, PM was associated with PE mostly by past malaria infection in placental histological grading^20^. In Senegal, histological diagnosis was made but the study did not present data based on the histological grading system as done in Sudan and this current study^47^. Therefore, conclusions cannot be drawn on whether the association was based on only active infections, past infections or both. Other studies generally associated PM with HDP and not PE only^18,19^ as this present study has shown. Placental pathology was not specifically related to PM and other factors in these studies. Muehlenbachs *et al*.^18^, however showed elevated levels of sVEGFR-1, a PE biomarker, in primigravids with PM, hypertension or both. They concluded that this marker, associated with endothelial or placental dysfunction, may be under selective pressure during first pregnancies in malaria endemic regions. Our study has brought to bear the link between placental pathology, PM (active or past infections) and PE development. In the multiple comparison model, blood pressure at booking which is prior to PE diagnosis was not predictive of the disorder. Others have reported an inverse relationship between parasite density and blood pressure^18^. Similarly, a lack of association between PM and non-proteinuric hypertension has been reported^19^. In view of this, our investigation suggests that a compromised placenta plays a critical role in PE pathogenesis and this is particularly significant in primigravidae exposed to PM. Although over 90% of participants received at least one dose of IPTp, our study did not find any relationship between placental pathology, IPTp use and PE development. However, it has recently been shown that high uptake of IPTp improves birth weight^48^. Further studies are needed to ascertain if high uptake of IPTp reduces placental pathology and the risk of PE in malaria endemic regions.

### Limitations

Our study was a single site study that has given a snapshot of the possible pathological effects of PM during PE. We propose a longitudinal multicenter study to examine pathological variations of PM together with other risk factors to further understand the heterogeneity of PE.

## Conclusion

Our study has shown for the first time among Ghanaian women pathological effects of a PE placenta in women exposed to PM. This may be an important consideration in future management guidelines for PE in malaria endemic regions. Furthermore, mechanisms that may induce PM specific PE should be elucidated.

## Methods

### Study site

The study was conducted at the Department of Obstetrics and Gynaecology of the Korle-Bu Teaching Hospital (KBTH) in Accra, Ghana. KBTH is a tertiary hospital and a leading national referral centre and hosts the University of Ghana School of Medicine and Dentistry. The Department of Obstetrics and Gynaecology is the largest department in the hospital with a 275-bed capacity for obstetric care (~10,000 deliveries/year). It has the capacity and medical expertise to manage major obstetric complications hence serves as a pivot for obstetric referrals from the southern half of the country.

### Study design and target population

The study was carried out between January and December 2017. Pregnant women diagnosed with PE and presenting for delivery at the hospital were screened for inclusion into the study. Healthy pregnancies (non-PE) were concurrently screened for inclusion from the same hospital. Informed consent was obtained from each participant recruited. Case report forms were appropriately completed to capture both demographic and clinical information. Each participant was assigned a unique identification code prior to sampling. Laboratory data were collected at delivery and transported to the Pathology Department of the Korle-Bu Teaching Hospital for analysis. Both primigravidae and multigravidae within the age range of 18–45 years were enrolled into the study.

### Inclusion criteria for preeclampsia (PE)

Clinical PE was defined as pregnant woman who presented with a history of sustained hypertension (blood pressure ≥140 mmHg systolic or ≥90 mmHg diastolic at least 4 hours apart) with proteinuria (two readings of 1+ or higher on urinalysis) ≥20 weeks of gestation as diagnosed by an obstetrician. This definition is in accordance with the International Society for the Study of Hypertension in Pregnancy (ISSHP) for the diagnosis of clinical PE^3,49^.

### Inclusion criteria for healthy or non-preeclamptic pregnancy (non-PE)

This was defined as pregnant woman at ≥20 weeks of gestation with a history of sustained blood pressure <140 mmHg systolic or <90 mmHg diastolic) without proteinuria at time of enrolment as confirmed by a clinician/midwife and medical records.

### General exclusion criteria

Women with multiple gestation, pre-existing cardiovascular disease, chronic hypertension, diabetes, infections such as HIV and hepatitis, haematological and immunological disorders were excluded.

### Ethical considerations

All methods were carried out in accordance with relevant guidelines and regulations. Approval to conduct this study was obtained from the Institutional Review Board of the NMIMR (IRB# 00001276)) registered with OHRP (FWA 00001824) and the Ethical and Protocol Review Committee of the KBTH (KBTH/MD/G3/17). Demographic data and biological specimen were obtained following informed consent from selected participants. Participation was strictly voluntary with no restrictions if participant decided to opt out of study.

### Measure of exposure to placental malaria

Placental Malaria (PM) was defined as the presence of parasites in the placental blood and/or parasite pigment deposition in placental biopsies after delivery as previously described^18,32^.

### Placental tissue sampling and processing

To investigate the association between placental pathology and *Plasmodium falciparum* infection, placental tissues were sampled from PE and non-PE women after delivery. A portion of excised tissue was washed in physiological saline to remove excess blood and immediately placed in a well labelled container with 100 ml of 10% neutral buffered formalin for tissue fixation. This was to maintain tissue integrity and minimize the formation of formalin pigment in the tissue. Biopsy was immediately transported to the Department of Pathology, KBTH, where tissue processing was carried out. Fixative was changed from time to time until a clear fixative was obtained that ensured that the tissue was well fixed before processing. Processing of fixed tissue was carried out within 72 hours of collection.

### Placental tissue processing

Placental tissues were processed using Leica TP1020 (Leica Biosystems, Germany) tissue processor. Briefly, a 3 cm^3^ (3 × 1 × 1 cm) portion of the placental tissue was grossed and placed into a well labelled tissue processing cassette and fixed in 10% neutral buffered formalin for about an hour. The tissue portion in the cassette was dehydrated in ascending grades of ethanol (60%, 70%, 80% and 90%) for 90 minutes. This was then placed in three changes of absolute ethanol to achieve full dehydration. After dehydration, tissue was cleared in two changes of xylene and transferred into molten paraffin wax at a melting temperature of 56 °C. Infiltration was carried out for 2 hours. For paraffin embedding, Leica EG1150, (Leica Biosystems, Germany) modular tissue embedder was used to form tissue blocks.

### Placental tissue sectioning and staining

Placental tissue block was trimmed at 10 microns and placed on ice to cool, after which 3 microns thick of tissue was sectioned into ribbons using a rotary microtome (Leica RM2235, Leica Biosystems, Germany). Ribbons were floated onto a protein-free water bath (Boekel Scientific 14793, USA) at a temperature of 50 °C. Two floating ribbons from each placental tissue were mounted onto well labelled grease-free frosted end glass slides. Subsequently, the slides were air dried and heat fixed on a hot plate. Placental tissue sections mounted on each slide were stained using standard haematoxylin and eosin (H&E) stains and Giemsa respectively. The H & E staining was carried out to differentiate cellular components of placental tissues according to Mayer’s staining protocol^50^. Briefly, sections were deparaffinized in xylene, rehydrated in absolute alcohol, conditioned in varying gradients of alcohol and stained with haematoxylin. Sections were counter stained in eosin, dehydrated, cleared and mounted with a xylene based mounting medium (DPX 44581, Sigma). Similar procedure was carried out for Giemsa staining.

### Microscopic examination of histologically stained placental tissues

Slides were blindly examined using a standard light microscope and a histological grading system adapted from Muehlenbachs and group^51^. Presence or absence of malaria parasites, hemozoin, inflammation and unique placental changes were recorded under x4, x10, x20 or x40 using the Olympus BX 43 microscope. Images were captured on the cellSens standard software using d27 camera. Infection state was classified into four groups namely acute infection (presence of parasites and absence of hemozoin), chronic infection (presence of parasites and significant amount of hemozoin in fibrin or macrophages), past infection (absence of parasites with presence of hemozoin in fibrin or macrophages) and no infection (absence of both parasites and hemozoin in fibrin or macrophages)^32,51^. Unique pathologies observed during the examination of the placenta were also reported.

### Statistical analysis

Data were analysed using GraphPad prism version 6 (California, USA) and R version 3.5.1 (R Development Core team). Means were compared between two continuous variables using Student’s t-test. One-way analysis of variance (ANOVA) test was used to compare means for more than two continuous variables. Pair-wise post-hoc comparisons were done using Dunn’s multiple comparison test. Categorical data was analysed using Chi-square test or Fisher’s exact test for association. Univariate and multivariate binary logistic regression analyses were carried out to predict the risk of PE. For all analyses, *P* < 0.05 was considered statistically significant.

## Supplementary information

Supplementary Information.

## Data Availability

The data generated during the current study is included in this article and its supplementary files.
