# Supplementary material for: Histopathological lesions and exposure to Plasmodium falciparum infections in the placenta increases the risk of preeclampsia among pregnant women
Source: Sci Rep. 2020 May 19;10:8280. doi: 10.1038/s41598-020-64736-4 (PMC7237676; doi:10.1038/s41598-020-64736-4)
Supplement: Supplementary file 1 — Supplementary Information. [file 41598_2020_64736_MOESM1_ESM.docx]

**Histopathological lesions and exposure to *Plasmodium falciparum* infections in the placenta increases the risk of preeclampsia among pregnant women**

Dorotheah Obiri^1,2*^, Isaac Erskine^3^_,_ Daniel Oduro,^2,4,5^, Kwadwo Asamoah Kusi^1,2^, Jones Amponsah^2^, Ben Gyan^2^, Kwame Adu-Bonsaffoh^6^, Michael Ofori^1,2*^

**Supplementary Figure S1. Relationship between placental pathology, maternal and delivery outcomes in non-PE and PE pregnancies.** (a) Placental pathology (%) in primigravid and multigravida women. (b) delivery age in normal (white boxplot) and pathologic placentae (dotted boxplot). Whiskers are presented as maximum and minimum values with medians represented by the horizonal lines across the boxes (c) birth weight (mean ± SD) in normal and pathologic placentae. ^*^P < 0.05, ^**^P < 0.01, ^***^ P < 0.001, ^****^ P < 0.0001.

**Supplementary Figure S2. Relationship between maternal and delivery outcomes in non-PE and PE pregnancies with placental malaria**. (A) Gestational age at delivery (mean ± SD) in women with no infection (grey dotted circles), active infection (inverted triangles) and past infection (dark circles) (B) Birth weight (mean ± SD) in women with no infection (plain circles), active infection (dark squares) and past infection (grey triangles). ^*^ (*P* < 0.05), ^***^ (*P* < 0.001).

**Supplementary Table S1. Placental pathology, maternal factors and the risk for preeclampsia**

| **Variable** | **OR (95 % CI)** | ***P*-value** | **AOR (95 %CI)** | ***P*-value** |
| --- | --- | --- | --- | --- |
| **Placental Pathology** |  |  |  |  |
| **No** | Ref | Ref | Ref |  |
| **Yes** | 2.2 (1.1 – 4.6) | **0.027** | 2.8 (1.2 – 6.4) | **0.016** |
| **Specific Pathology** |  |  |  |  |
| **Mixed Pathology** | 5.76 (0.7 – 50.7) | 0.115 | - | **-** |
| **Infarction** | - | - | - | **-** |
| **Calcification** | 0.41 (0.2 – 1.1) | 0.067 | - | **-** |
| **Atherosis** | 2.16 (0.19 – 24.4) | 0.534 | - | **-** |
| **Accelerated maturation** | - | - | - | **-** |
| **Syncytial Knots** | 10.1 (2.2 - 47.3) | **0.003** | - | **-** |
| **1^st^ visit SBP (mmHg)** | 1.04 (1.01 – 1.07) | **0.010** | 1.0 (1.0 - 1.1) | **0.035** |
| **1^st^ visit DBP (mmHg)** | 1.04 (1.0 0– 1.07) | **0.042** | 1.0 (0.9 – 1.1) | 0.86 |
| **Gravidity** |  |  |  |  |
| **Multigravid** | Ref | Ref | Ref |  |
| **Primigravid** | 3.0 (1.5 – 6.3) | **0.003** | 4.5 (2.0 – 10.7) | **<0.0001** |

Univariate and multivariate analysis showing factors associated with an increased risk of PE. Data presented as odds ratio (OR), adjusted odds ratio (AOR) with confidence interval (CI). SBP and DBP = systolic and diastolic blood pressure respectively. *P* < 0.05 was statistically significant.

**Supplementary Table S2. *Plasmodium falciparum* exposure in non-preeclamptic and preeclamptic placentas**

| **Bulmer score** | **Non-PE (%)** | **PE (%)** | **Total (%)** | ***P*-value** |
| --- | --- | --- | --- | --- |
| **No Infection** | 38 (55.1) | 10 (15.6) | 48 (36.1) | **< 0.0001** |
| **Acute** | 5 (7.2) | 11 (17.2) | 16 (12.0) |  |
| **Chronic** | 20 (29.0) | 28 (43.8) | 48 (36.1) |  |
| **Past Infection** | 6 (8.7) | 15 (23.4) | 21 (15.8) |  |
| **Total** | 69 | 64 | 133 |  |

Data presented as proportions between non-preeclamptic group (non-PE) and preeclamptic group (PE). *P*-value obtained by Fisher’s exact test.
